# Next-generation sequencing of the CHO cell transcriptome

**DOI:** 10.1186/1753-6561-5-S8-P6

**Published:** 2011-11-22

**Authors:** Jennifer Becker, Christina Timmermann, Tobias Jakobi, Oliver Rupp, Rafael Szczepanowski, Matthias Hackl, Alexander Goesmann, Andreas Tauch, Nicole Borth, Johannes Grillari, Alfred Pühler, Thomas Noll, Karina Brinkrolf

**Affiliations:** 1 Department of Cell Culture Technology, Center of Biotechnology (CeBiTec), Bielefeld University, 33615 Bielefeld, Germany; 2 Bioinformatics Resource Facility, Center of Biotechnology (CeBiTec), Bielefeld University, 33615 Bielefeld, Germany; 3 Department of Genome Research and Systems Biology, Center of Biotechnology (CeBiTec), Bielefeld University, 33615 Bielefeld, Germany; 4 Department of Biotechnology, University of Natural Resources and Applied Life Sciences Vienna, 1180 Vienna, Austria

## 

Since 1957 Chinese hamster ovary (CHO) cells are used for *in vitro* cultivation as they require assimilable low sustenance [1]. Today, CHO cell lines represent the most commonly used mammalian expression system for the production of therapeutic proteins and are considered as the mammalian equivalent of *E. coli* in research and biotechnology [2]. The production of biopharmaceuticals in CHO cells is superior to protein production in bacteria, because mammalian cell lines procure complex folding and post-translational modifications like glycosylation. However, contrary to the increasing importance in biotechnology and industry, comprehensive genome and transcriptome information of CHO cell lines is still rare.

In this study, the pyrosequencing technology from 454 Life Sciences and a novel assembly approach for cDNA sequences were used to achieve a major step forward towards unraveling the transcriptome of CHO cells.

CHO cDNA samples derived from different CHO cell lines and growth conditions were used for the generation of 1.84 mill. high quality sequencing reads with an average read length of 373 nt summing up to 603 Mb data. Assembly of the sequencing data resulted in 41,039 contiguous sequences. These contigs were grouped by the Newbler software into 36,383 isotigs and 28,039 isogroups.

Taxonomical classification and comparison to the *Mus musculus* transcriptome demonstrated the actual quality of the CHO cell line sequences.

Metabolic pathways of the central carbohydrate metabolism and biosynthesis routes of sugars used for protein *N*-glycosylation were reconstructed from the transcriptome data. All relevant genes representing major steps in the *N*-glycosylation pathway and the central metabolism of CHO cells were detected. Only fructose-1,6-bisphosphatase (3.1.3.11) and 6-phosphogluconolactonase (3.1.1.31) were not identified within the pentose phosphate pathway.

**Figure 1:**
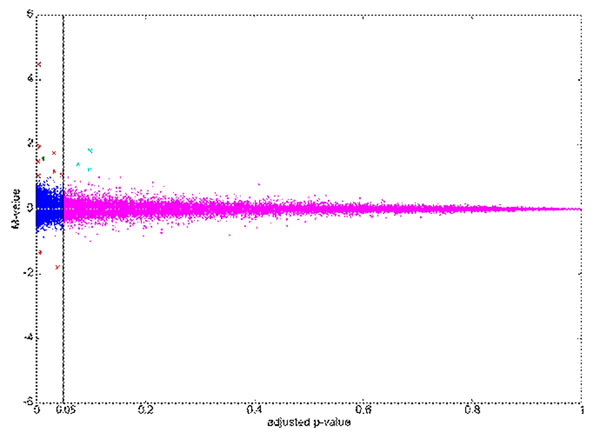
Self-Self Hybridization experiment of the customized CHO microarray. The dye ratio (M-value) is plotted against the adjusted p-value (student´s t-test, FDR controlled, α=0.05). The dye ratio was calculated using the mean intensity values (A-value) of all probes belonging to one transcript from four microarray replicates. Different thresholds were used as evidence for quality: p > 0.05, M= - 1< M < 1 (pink); p ≤ 0.05, -1 < M < 1 (dark blue); p ≤ 0.05, M ≤ -1 || M ≥ 1, A < 6 (red); p ≤ 0.05, M ≤ -1 || M ≥ 1, A ≥ 6 (green); p > 0.05, M ≤ -1 || M ≥ 1 (light blue).

The newly sequenced CHO cell line transcriptome was the basis for the design of a customized CHO microarray. Contig sequences were used for the design of 94.580 probes. The designed probes cover 31,905 splice variants of CHO transcripts. With a Self-Self Hybridization experiment (Figure 1) the functionality of the probes was demonstrated. This experiment was performed with the same RNA as which was used for sequencing. Half of this RNA was labeled with Cy3, the other half with Cy5. For this study the dye intensity, the dye ratio and the adjusted p-value (student´s t-test, FDR controlled, α=0.05) of four microarray replicates were analyzed. It is expected, that the two labeled RNA samples bind equally to the probe, if a transcript is expressed (Figure 1: pink, dark blue, red, light blue). Only the probes for one transcript could be rejected, because of their dysfunctionality (Figure 1, green).

This CHO microarray is now available for further experiments and will support transcriptional analysis of CHO cells under process conditions for cell line and process optimization. It was used already used successfully for a gene expression study of CHO DP-12 cells cultivated under sodium butyrate treatment [3].
